# Bone Preservation for Replacement of Lateral Incisors: A Case Report

**DOI:** 10.1155/crid/2445685

**Published:** 2026-07-13

**Authors:** Luigi Laino, Roberto Ciarlantini, Mark Wertheimer

**Affiliations:** ^1^ Assistant Oral Pathology Department, Federico II University, Naples, Italy, unina.it; ^2^ Private Practice of Orthodontics, Recanati, Italy; ^3^ Private Practice of Orthodontics, Johannesburg, South Africa

**Keywords:** alveolar bone maintenance, alveolar ridge resorption, orthodontic preprosthetic treatment, skeletal anchorage, tooth agenesis

## Abstract

An adolescent female patient presenting with bilateral agenesis of upper lateral incisors was treated with opening of spaces for later implant insertion at adult age. During the interim period after completion of orthodontic treatment and ultimate implant placement, two horizontally orientated miniscrews were inserted in the edentulous areas to prevent alveolar ridge resorption. After observation and measurement of CBCT follow‐up images, it can be argued that this new technique was effective in maintaining alveolar bone in this patient. Although bone grafts before implant insertion were not avoided, their difficulty was diminished and less operator‐dependent. This enhanced predictability and clinical success.

## 1. Introduction

Congenitally missing lateral incisors are not uncommon, with reports of incidence varying between 1% and 7.6% according to different publications [1, 2]. A variety of options exist for the treatment of patients with missing lateral incisors. These include canine substitution with the canine tooth ultimately in the lateral incisor position [3, 4], or a prosthetic solution. Prosthetic solutions include the placement of resin‐bonded bridges [5–7], ideally with little or no preparation of the abutment teeth, autotransplantation [8], or implants [5, 6, 9].

Long‐term follow‐up controls regarding periodontal conditions and aesthetic appearance are better in space closure cases than space opening cases [10, 11]. In addition, this approach allows for harmonious growth and maturation of the dentition and alveolar processes without the documented problems related to infraposition of the implant over time [12, 13].

Scientific evidence dictates that implants should be placed as late as possible in order to prevent the negative sequelae of infraposition [14, 15]. However, if implants are planned, ideally, they should be placed further posteriorly [16]. If implants are planned for the site of the missing lateral incisor, then it is necessary to maintain bone in the future implant site until it is appropriate to place the implants, as loss of bone has been noted by Uribe and coworkers [17, 18].

Ciarlantini and Melsen have proposed and published an approach to preserve bone in the future implant site using a horizontally placed mini‐implant [19–22]. This approach has evinced some success, which has also been supported by various other studies [23–26]. This case report is the first in the literature showing radiological 3D data, long‐term follow‐up until implant insertion and comparative observation between loaded and nonloaded miniscrews. However, being a case report, no definitive conclusion can be reached. A further 3D longitudinal study by the authors is planned to demonstrate the validity of this technique from a more evidence‐based perspective.

## 2. Description of the Case

**Figure 1:**
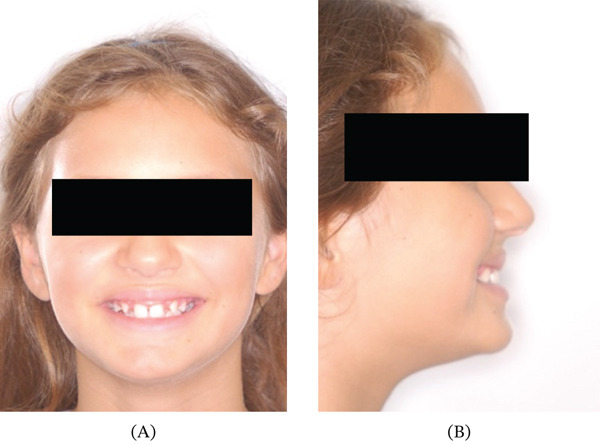
(A, B) Extraoral appearance of the patient before orthodontic treatment.

An 11‐year‐old adolescent patient presented in the mixed dentition with bilateral agenesis of maxillary lateral incisors. The sagittal and vertical relationships of the face, as well as incisor exposure during smiling, were all within the normal range. The orthodontic evaluation revealed a skeletal Class I pattern (Figure 1A,B).

Intraoral examination of the maxillary arch revealed mesially rotated first molars and a retained left deciduous second molar. The canines had drifted mesially, although there was space between them and the central incisors due to the missing lateral incisors. A small midline diastema was present.

There was mild crowding of the mandibular incisors, with the lower left second premolar presently unerupted.

With respect to the interarch relationships, the canines displayed a cusp‐to‐cusp relationship on the right and a Class I relationship on the left.

The molars displayed a neutrocclusion due to the mesial rotation of the upper molars.

**Figure 2:**
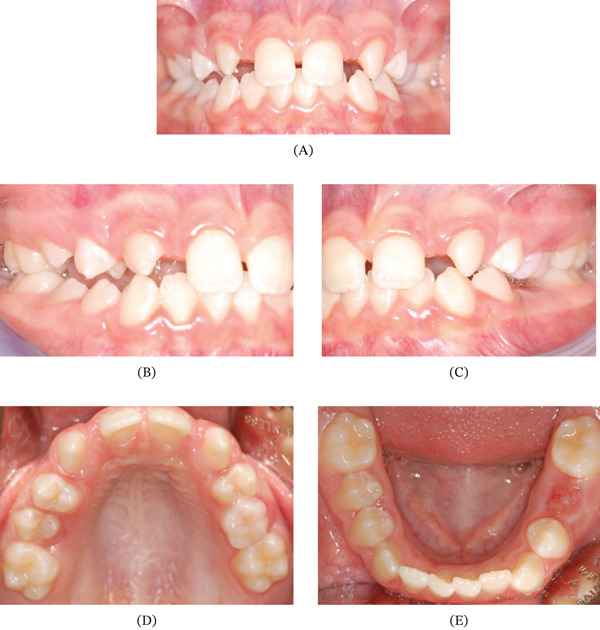
(A–E) Intraoral appearance and occlusion of the patient before orthodontic treatment.

The overjet was normal, and the overbite increased with the maxillary incisors covering 50% of the mandibular incisors (Figure 2A–E).

### 2.1. Treatment Plan

The final treatment plan decided upon involved the opening of space for prosthetic rehabilitation of the 12 and 22, ultimately with the placement of implant‐supported prostheses (Table 1). Upper and lower fixed orthodontic appliances were placed. Brackets on upper canines were bonded with a slight mesial inclination in order to obtain distal uprighting of their roots to provide sufficient interradicular space for implants at a later stage in adulthood.

**Table 1:** Timeline for the treatment of the patient.

Age	Treatment
11–13 years	Orthodontic treatment
13–21 years	Waiting period after insertion of miniscrews
21–21.4 years	Implants insertion, bone graft, and osseointegration waiting period
21.4–21.8 years	Soft tissue healing after implants exposure, connective tissue graft, and provisional crowns insertion
21.8–21.9 years	Final impressions and definitive crowns cementation
21.9–24.9 years	No treatment
24.9 years	Follow‐up records

**Figure 3:**
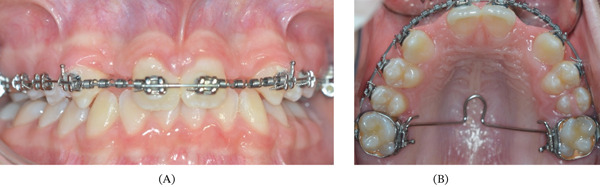
(A, B) Orthodontic treatment progress.

A transpalatal bar was placed and activated to derotate the upper first molars and gain slight expansion of the upper arch (Figure 3A,B).

**Figure 4:**
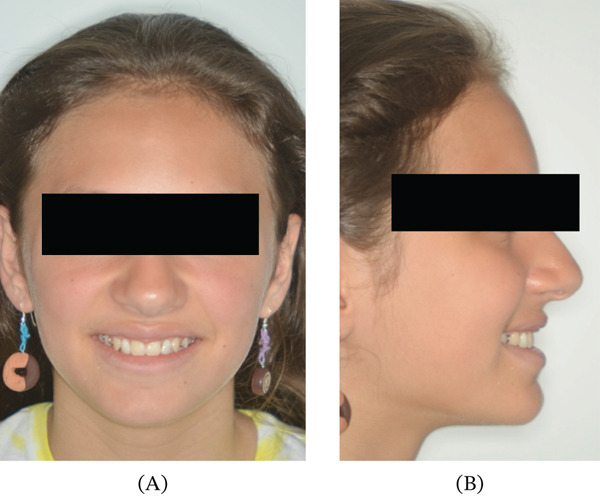
(A, B) Extraoral appearance at the end of orthodontic treatment.

After 2 years of orthodontic treatment, when the patient was 13 years old, Class I canine and molar relationships were achieved bilaterally, and the fixed orthodontic appliances were removed in March 2013. A harmonious soft tissue profile and smile were evident (Figure 4A,B).

**Figure 5:**
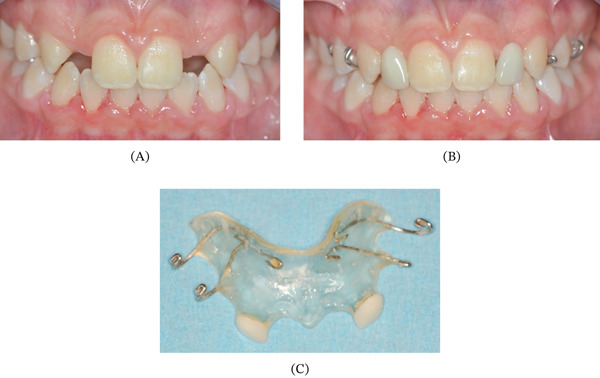
(A–C) Removable appliance with two pontics.

On the day of debonding, the patient received a removable upper retention appliance with two temporary resin pontics to substitute for the missing upper lateral incisors (Figures 5A‐C). In the lower arch, a fixed lingual bonded retainer was applied from 33 to 43.

**Figure 6:**
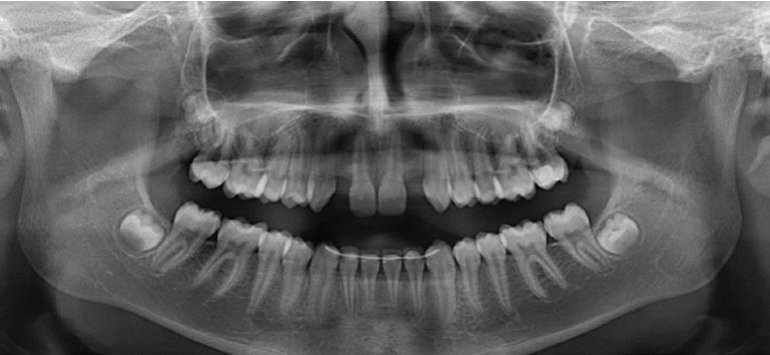
Panoramic radiograph.

**Figure 7:**
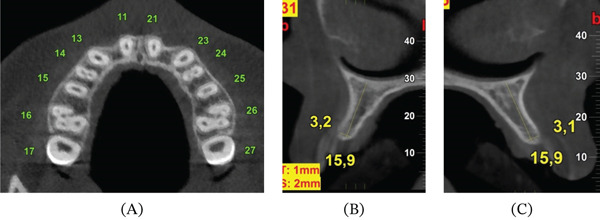
(A–C) CBCT images at the beginning of the retention period.

At that time, a panoramic radiograph and CBCT scan were taken. The quantity of bone and interradicular space was sufficient for future implant placement in the 12 and 22 sites at adult age (Figures 6 and 7A‐C).

Two orthodontic miniscrews normally used as temporary anchorage devices (TADS), were inserted horizontally at the level of the middle third of the interradicular space in the sites of the 12 and 22. This is in accordance with the protocol described by Ciarlantini and Melsen [21], to maintain bone morphology and prevent resorption of the alveolar process during the period preceding implant placement at a more appropriate age.

**Figure 8:**
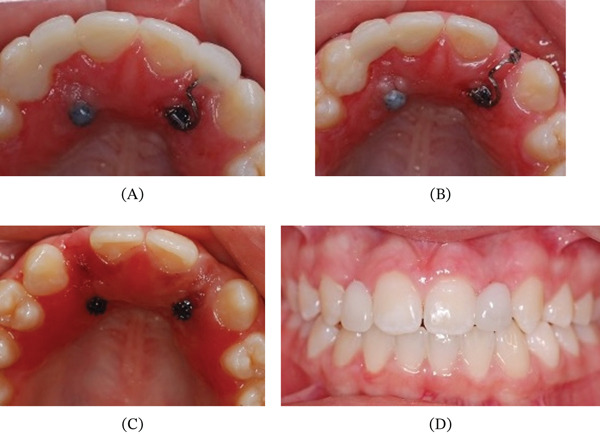
(A–D) Intraoral images after insertion of the miniscrews.

**Figure 9:**
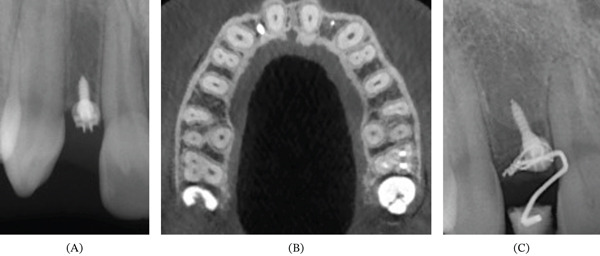
(A–C) Radiographs after insertion of the miniscrews.

On the right, a disilicate Maryland bridge was cemented for temporary substitution of the 12, whereas in the edentulous area on the left in the site of the 22, a 0.021x0.025 SS sectional wire was inserted and ligated into the slot of the miniscrew [21]. A pontic in place of the 22 was attached to this wire. This allowed for assessment of any differences in miniscrew behavior based on whether it had a pontic attached or not. Intraoral images (Figures 8A‐D) and radiographs (Figures 9A‐C) were taken after application of the pontics, and annually thereafter.

This was in place from 2013 until 2018, when the pontic was removed from the left miniscrew and replaced with a Maryland bridge at the request of the patient.

**Figure 10:**
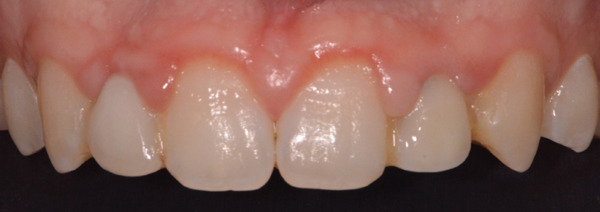
Photograph at age 21.

**Figure 11:**
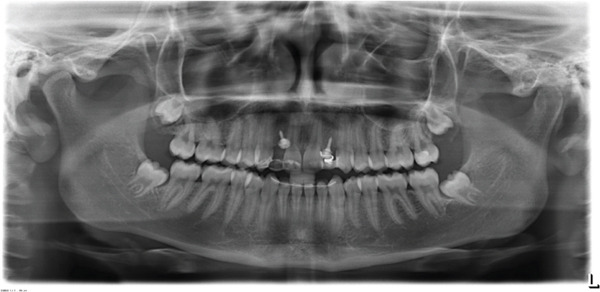
Panoramic radiograph.

**Figure 12:**
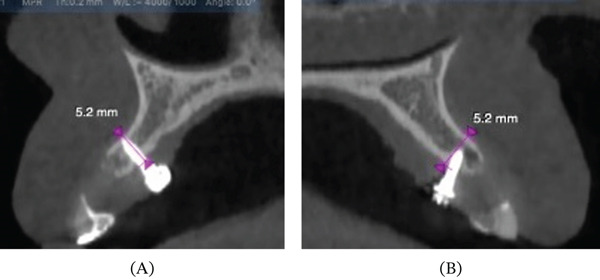
(A, B) CBCT 4 years and 6 months after placement of miniscrews.

Eight years later at 21 years of age, photographs (Figure 10), a radiograph (Figure 11), and a CBCT (Figure 12A, B) were taken to evaluate bone thickness in both edentulous areas. It can be noted that both the cortical plate and medullary bone had been maintained. The patient was scheduled for implant insertion in the edentulous 12 and 22 sites.

**Figure 13:**
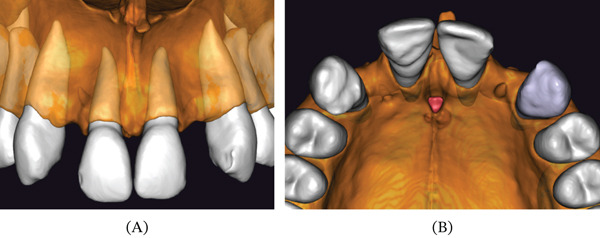
(A, B) 3D model from CBCT images before implant insertion.

In June 2021 a CBCT was taken at removal of the miniscrews, and prior to placement of implants (Figure 13A,B).

**Figure 14:**
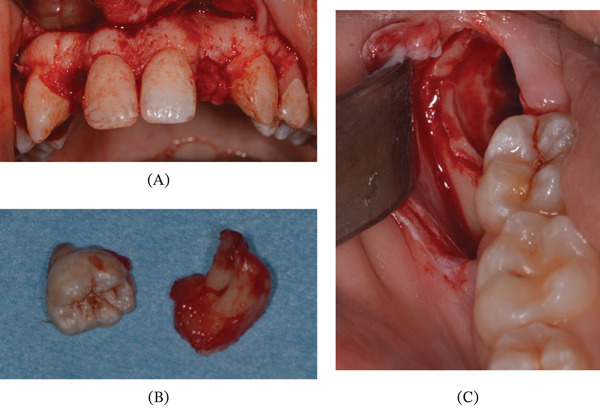
(A–C) Extraction of wisdom teeth and implant site preparation.

The Maryland bridges and miniscrews were removed, and a full‐thickness flap was raised for implant site preparation. To increase the convexity of the alveolar process on the labial aspect of the edentulous areas in the 12 and 22 sites, the 38 and 48 were extracted, and bone was harvested near the external oblique ridge of the mandible for autologous bone grafting onto the labial aspect of the implants after their insertion. (Figure 14A–C).

**Figure 15:**
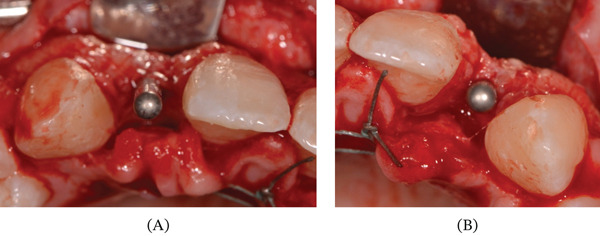
(A, B) Implant placement.

**Figure 16:**
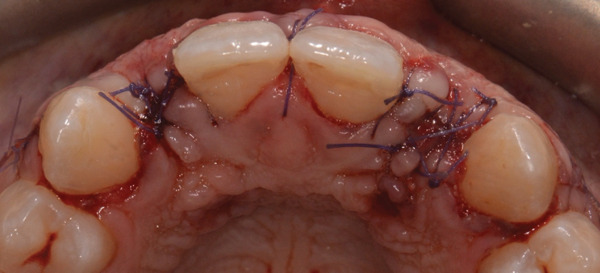
Closure of surgical flaps.

**Figure 17:**
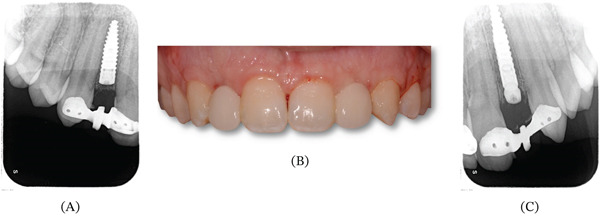
(A–C) Maryland bridges were recemented after implant placement.

Two 3.3‐mm diameter Straumann conic implants were placed at the existing bone level (Figure 15A,B). On the labial aspect of each of the implant sites, the bone graft was performed using autologous bone combined with bovine bone (Bio‐Oss), as well as membranes being applied to cover the grafts. The flap was closed and sutured (Figure 16), and Maryland bridges were recemented. (Figure 17 A‐C).

In August 2021, bleaching was carried out to address the color of the dentition at the request of the patient.

**Figure 18:**
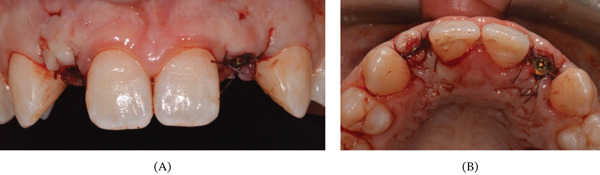
(A, B) Implant exposure and insertion of healing screws.

In October 2021, 4 months after implant placement, the implants were exposed, and an epithelial connective tissue graft harvested from the palate was applied buccally to the implants to increase the thickness of the soft tissue. Three‐millimeter healing abutments were placed. (Figure 18A,B).

**Figure 19:**
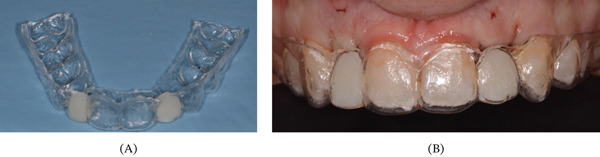
(A, B) Essix retainer.

During the following 3‐week healing period, the patient wore an Essix vacuum‐formed retainer with two pontics inserted into it. (Figure 19A,B).

**Figure 20:**
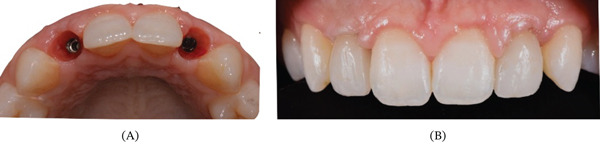
(A,B) Provisional crowns.

Provisional screw‐retained crowns were placed to develop the soft tissue in the implant sites in Nov 2021 (Figure 20A, B).

**Figure 21:**
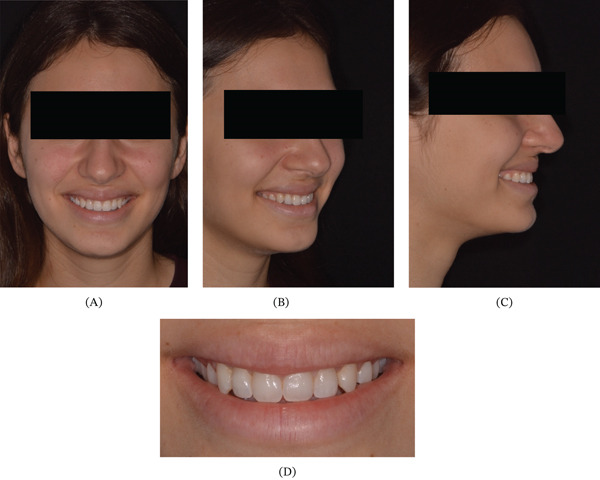
(A–D) Extraoral photographs.

**Figure 22:**
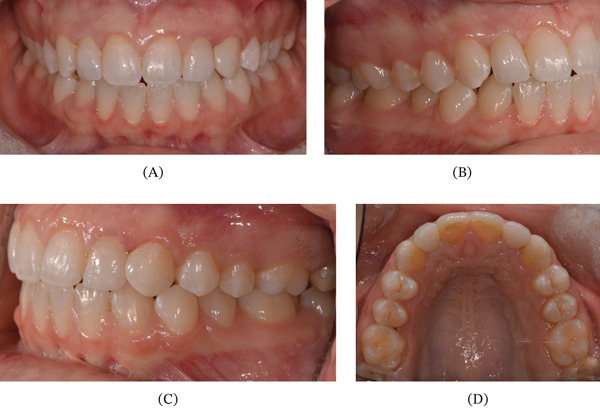
(A–D) Intraoral photographs.

After approximately 3 months, the soft tissues had matured around the provisional crowns, and definitive impressions were made. Two customized zirconia abutments were prepared, and definitive zirconia crowns were cemented in March 2022 after customization of the buccal surface. The choice of a cemented instead of a screw‐retained crowns was necessary because the screws caused a gray shadow with an unaesthetic appearance of the zirconia crowns. Extraoral (Figure 21A–D) and intraoral views of the patient after definitive crown cementation (Figure 22A–D).

**Figure 23:**
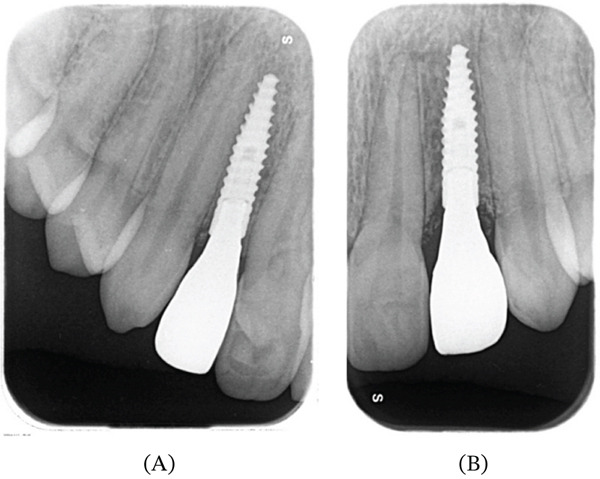
(A, B) Periapical radiographs.

**Figure 24:**
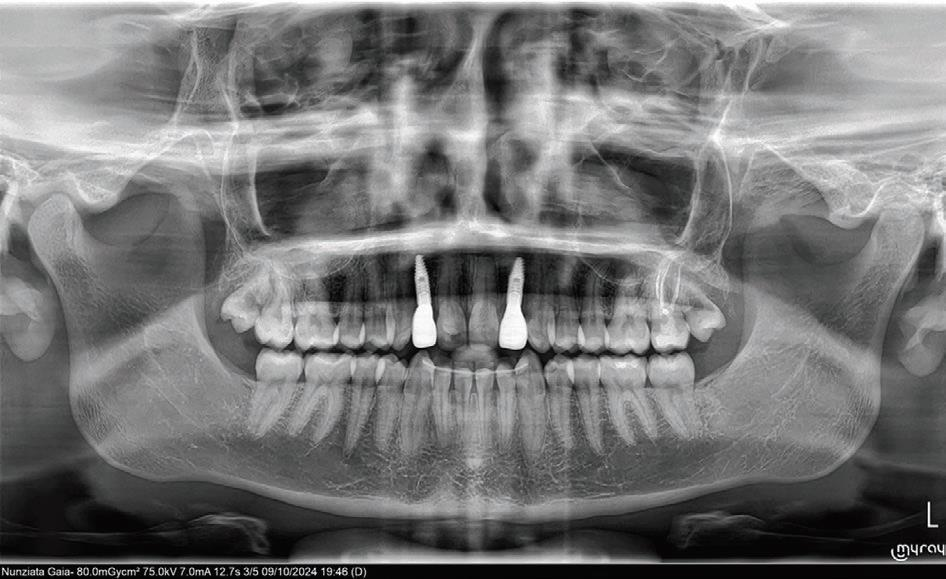
Panoramic radiograph.

**Figure 25:**
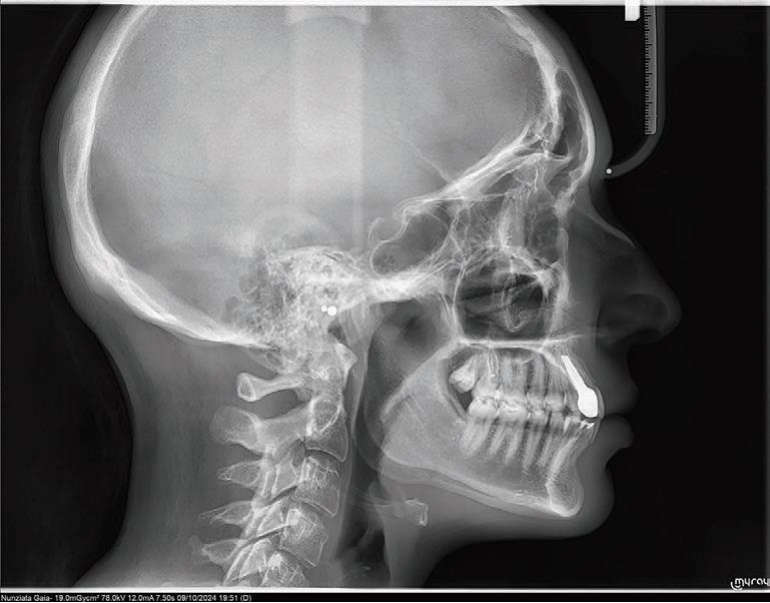
Lateral cephalometric radiograph.

**Figure 26:**
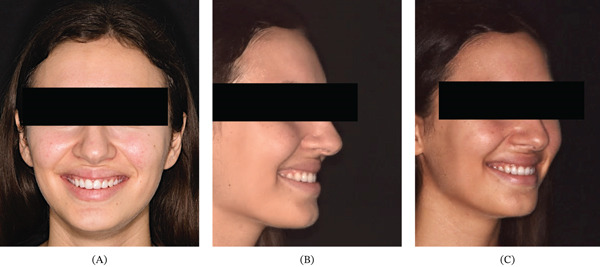
(A–C) Extraoral images.

**Figure 27:**
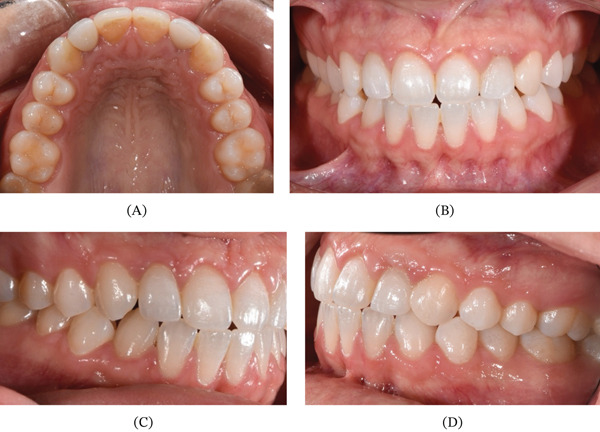
(A–D) Intraoral images.

In September 2024, at a follow‐up 3 years after implant placement, radiographic controls (Figure 23A,B; Figures 24 and 25), and both extraoral (Figure 26A–C) and intraoral images (Figure 27A–D) were taken. The implants were stable with good healing of the bone around the implants being evident. The periodontal tissues were healthy and aesthetically satisfactory with a good appearance of the prosthetic rehabilitation.

The CARE reporting guidelines were used in describing this case report [27] and the patient allowed personal data processing, and informed consent was obtained from her.

## 3. Results

The miniscrew retained pontics were well accepted with no complications or discomfort during the interim waiting period. The miniscrews maintained the bone in the future implant sites during the period following completion of orthodontic treatment, prior to the ultimate placement of the implants. The final records taken 3 years postcompletion of treatment revealed a very pleasing aesthetic result. The bone and soft tissue morphology surrounding the implants were also excellent.

**Figure 28:**
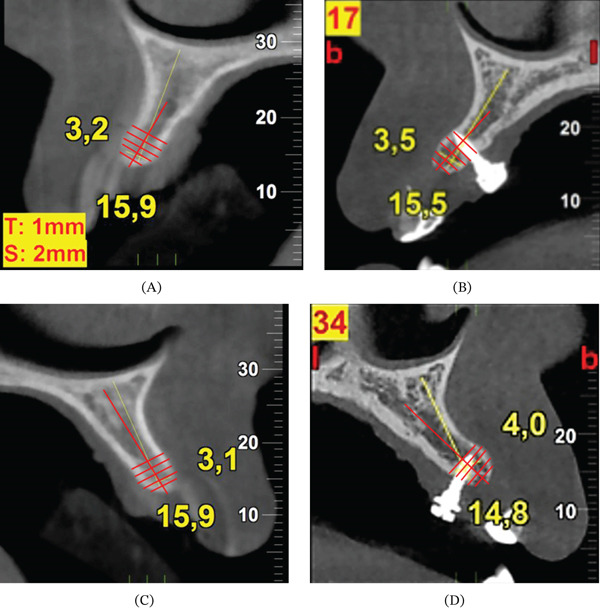
(A–D) Comparative CBCT images (measured).

**Figure 29:**
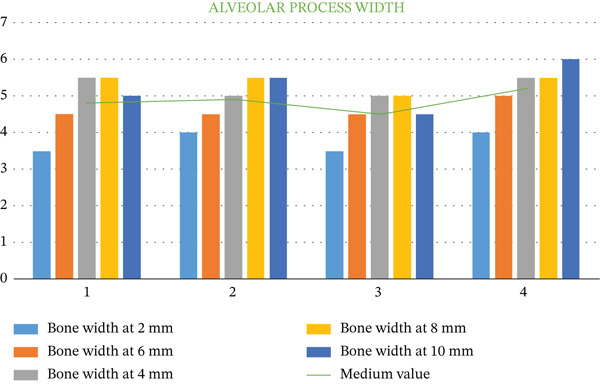
Alveolar process widths.

Measurements on CBCT images (Figure 28A–D) according to the method described by Uribe and coworkers [17, 18] were undertaken to observe maintenance of the alveolar ridge anatomy over the years. The values of the measurements in Table 2 and Figure 29, reveal no significant loss of bone volume.

**Table 2:** CBCT measurements.

	T0 (12) 1	T0 (22) 2	T1 (12) 3	T1 (22) 4
Bone width at 2 mm	3,5	4	3,5	4
Bone width at 4 mm	4,5	4,5	4,5	5
Bone width at 6 mm	5,5	5	5	5,5
Bone width at 8 mm	5,5	5,5	5	5,5
Bone width at 10 mm	5	5,5	4,5	6
Medium value	4,8	4,9	4,5	5,2

The restorative treatment, and in particular the morphology and emergence profile of the crowns, as well as the periodontal grafting procedures, ensured the health and appearance of the soft tissue surrounding the implant‐supported crowns.

The records taken 3 years after placement of the implants did not exhibit any significant evidence of infraposition, although this might still occur. Nevertheless, continuing evaluation is recommended.

## 4. Discussion

While waiting for the completion of growth, temporary replacement of missing teeth with a removable plate or a bonded bridge is necessary. The disadvantage of a removable plate is that it demands compliance. Furthermore, the roots of the teeth adjacent to the future implant site may start impinging on the space prepared and being preserved for the implants [28]. In addition, the continuous coverage of the palatal mucosa over the years might result in disepithelization of the mucosa [29]. On the other hand, adhesive bridges often have compromised retention due to the clinical crowns in young patients being short. This might sometimes necessitate invasive procedures on healthy teeth to improve retention.

A miniscrew with a pontic for temporary substitution of the upper lateral incisor is utilized not only for alveolar bone maintenance, but also to overcome some of the problems posed by classical temporary replacement devices.

Moreover, miniscrews behave in a similar way to conventional implants. Bone is continuously deformed during physiologic dynamic loading with implants or miniscrews, which are more rigid than bone. The microstrain created at the bone/implant interface is sufficient to stimulate osteoblastic activity, which maintains bone density over time [30]. This increased bone density at the bone/implant interface in comparison with adjacent bony areas has been confirmed in various publications [31].

The first publication advocating the use of a miniscrew inserted perpendicularly into the alveolar process to support a temporary tooth in the case of a missing maxillary lateral incisor was published in 2007 [32]. Afterwards, other case reports were published in the literature advocating a similar protocol of vertical insertion of the miniscrew [33–36].

However, Kokich pointed out that a miniscrew inserted vertically in a growing patient would be problematic, as it might lead to a vertical angular bony defect due to penetration of the periosteum, thereby compromising normal bone development [37].

It is also noteworthy that thin miniscrews with a diameter of less than 1.6 mm may not be strong enough to resist fracture. The possibility of a mini‐implant failure and bone resorption in the aesthetic zone may lead to further complications [38].

To avoid such complications, Ciarlantini and Melsen [21] suggested the horizontal bicortical insertion of miniscrews as bone maintainers, at the occlusal third of the length of the roots of adjacent teeth, in the edentulous area. This horizontal insertion is also useful in cases of premolar agenesis, where both the buccal and lingual cortical plates are easily engaged, whereas with vertical insertion, this is not possible.

In order to select the correct length and inclination of the miniscrew for insertion, a surgical guide is recommended for an optimal result. This technique has been successfully applied in many patients and will be published in the future as a longitudinal study.

Conventional techniques for surgical implant placement and prosthetic rehabilitation were used in this patient, with a surgical flap allowing for clear vision of underlying bone and insertion of both a heterologous and autologous bone graft. The heterologous bone graft (Bio‐Oss) displays slow resorption and is useful when implant insertion is postponed for a prolonged period. The autologous bone graft increases the success of osteointegration.

## 5. Conclusions

The placement of a horizontally orientated miniscrew in the site of a missing maxillary lateral incisor is a viable option to maintain bone for later placement of an implant. Informed consent following a comprehensive explanation is recommended since infraposition in the future with an implant may occur.

## Author Contributions

Dr. Luigi Laino treated the patient. Dr. Roberto Ciarlantini wrote the manuscript. Dr. Mark Wertheimer contributed to the writing of the manuscript, correction of text, images, and references.

## Funding

No funding was received for this manuscript.

## Conflicts of Interest

The authors declare no conflicts of interest.

## Data Availability

The data that support the findings of this study are available on request from the corresponding author. The data are not publicly available due to privacy or ethical restrictions.
